# Metabolic engineering of *Saccharomyces cerevisiae* to produce a reduced viscosity oil from lignocellulose

**DOI:** 10.1186/s13068-017-0751-y

**Published:** 2017-03-20

**Authors:** Tam N. T. Tran, Rebecca J. Breuer, Ragothaman Avanasi Narasimhan, Lucas S. Parreiras, Yaoping Zhang, Trey K. Sato, Timothy P. Durrett

**Affiliations:** 1 Department of Biochemistry and Molecular Biophysics, Kansas State University, 141 Chalmers Hall, Manhattan, KS 66506 USA; 2 DOE Great Lakes Bioenergy Research Center, University of Wisconsin-Madison, Madison, WI 53726 USA

**Keywords:** Acetyl-TAGs, *Saccharomyces cerevisiae*, AFEX corn stover hydrolysate, Metabolic engineering

## Abstract

**Background:**

Acetyl-triacylglycerols (acetyl-TAGs) are unusual triacylglycerol (TAG) molecules that contain an *sn*-3 acetate group. Compared to typical triacylglycerol molecules (here referred to as long chain TAGs; lcTAGs), acetyl-TAGs possess reduced viscosity and improved cold temperature properties, which may allow direct use as a drop-in diesel fuel. Their different chemical and physical properties also make acetyl-TAGs useful for other applications such as lubricants and plasticizers. Acetyl-TAGs can be synthesized by *Ea*DAcT, a diacylglycerol acetyltransferase enzyme originally isolated from *Euonymus alatus* (Burning Bush). The heterologous expression of *Ea*DAcT in different organisms, including *Saccharomyces cerevisiae*, resulted in the accumulation of acetyl-TAGs in storage lipids. Microbial conversion of lignocellulose into acetyl-TAGs could allow biorefinery production of versatile molecules for biofuel and bioproducts.

**Results:**

In order to produce acetyl-TAGs from abundant lignocellulose feedstocks, we expressed *Ea*DAcT in *S. cerevisiae* previously engineered to utilize xylose as a carbon source. The resulting strains were capable of producing acetyl-TAGs when grown on different media. The highest levels of acetyl-TAG production were observed with growth on synthetic lab media containing glucose or xylose. Importantly, acetyl-TAGs were also synthesized by this strain in ammonia fiber expansion (AFEX)-pretreated corn stover hydrolysate (ACSH) at higher volumetric titers than previously published strains. The deletion of the four endogenous enzymes known to contribute to lcTAG production increased the proportion of acetyl-TAGs in the total storage lipids beyond that in existing strains, which will make purification of these useful lipids easier. Surprisingly, the strains containing the four deletions were still capable of synthesizing lcTAG, suggesting that the particular strain used in this study possesses additional undetermined diacylglycerol acyltransferase activity. Additionally, the carbon source used for growth influenced the accumulation of these residual lcTAGs, with higher levels in strains cultured on xylose containing media.

**Conclusion:**

Our results demonstrate that *S. cerevisiae* can be metabolically engineered to produce acetyl-TAGs when grown on different carbon sources, including hydrolysate derived from lignocellulose. Deletion of four endogenous acyltransferases enabled a higher purity of acetyl-TAGs to be achieved, but lcTAGs were still synthesized. Longer incubation times also decreased the levels of acetyl-TAGs produced. Therefore, additional work is needed to further manipulate acetyl-TAG production in this strain of *S. cerevisiae*, including the identification of other TAG biosynthetic and lipolytic enzymes and a better understanding of the regulation of the synthesis and degradation of storage lipids.

**Electronic supplementary material:**

The online version of this article (doi:10.1186/s13068-017-0751-y) contains supplementary material, which is available to authorized users.

## Background

Fossil-derived carbon represents the major source of the fuels and chemical products used by modern society. As this source is finite, and because combustion of fossil fuels contributes to climate change, alternate sustainable sources for energy and chemical precursors are being sought. Microbial conversion of renewable plant feedstocks into biofuels and commodity or specialty chemicals represents one strategy to replace our current dependence on fossil fuels.

The yeast *Saccharomyces cerevisiae,* which has long been used by the fuel ethanol industry, is considered a potential biocatalyst to convert sugars from lignocellulosic biomass into biofuels. *S. cerevisiae* displays robust tolerance to industrial conditions and is highly efficient at fermenting glucose. However, native *S. cerevisiae* cannot catabolize xylose, which can make up almost half of the total sugars in plant biomass [1]. Thus, *S. cerevisiae* has been genetically engineered and evolved to convert xylose into ethanol. This includes the introduction of fungal xylose reductase, xylitol dehydrogenase, and xylulokinase (XR-XDH-XK), to allow the conversion of xylose into xylulose-5-phosphate, which can then be converted into acetyl-CoA for catabolic or anabolic processes (reviewed recently in [2, 3]). Despite these genetic changes, engineered yeast still displays diauxic sugar consumption; glucose is preferentially consumed first, followed by xylose. In addition to ethanol, isobutanol [4–7], butanol [8], and fatty acid [9–12] biofuels have been generated from pure glucose and xylose by engineered *S. cerevisiae*, but not from sugars derived from lignocellulosic biomass.

**Fig. 1:**
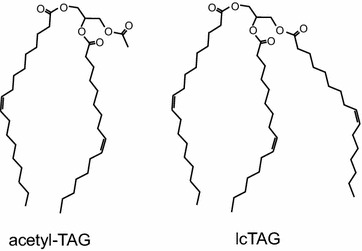
Acetyl-TAGs possess an *sn*-3 acetate group. Structures of representative acetyl-TAG and long-chain TAG (lcTAG) molecules

Another potential group of molecules that could be used not only as biofuel but also as a bioproduct for chemical upgrading are 3-acetyl-1,2-diacyl-glycerols (acetyl-TAGs), unusual triacylglycerol (TAG) molecules that possess an *sn*-3 acetate group (Fig. 1). The presence of the short acetate group rather than a long fatty acid means that acetyl-TAGs possess unique chemical and physical properties compared to regular long chain TAGs (hereon referred to as lcTAG; Fig. 1). For example, acetyl-TAGs possess lower viscosity and improved cold-temperature characteristics compared to other vegetable oils [13, 14]. Because the viscosities of most plant-derived triacylglycerols prevent their direct use in diesel engines [15], acetyl-TAGs could potentially be used as an improved low-viscosity straight vegetable oil biofuel. The higher oxygenated state of lipid-derived diesel replacements compared to conventional diesel results in lower emissions of particulates and other chemical pollutants [16], suggesting other environmental benefits to the use of these renewable fuels. In addition, with their *sn*-3 acetate group, acetyl-TAGs are structurally equivalent to semi-synthetically produced acetic acid esters of mono- and diglycerides (ACETEM) which can be used as food grade emulsifiers, lubricants, and non-phthalate-based plasticizers for polyvinyl chloride (PVC), and other plastic products [17]. In fact, tests of plastic films produced using ACETEM revealed that the vegetable oil-based plasticizers possessed better mechanical performance compared to conventional phthalate-based plasticizers [18].

While acetyl-TAGs are naturally found in the seeds of a number of different plant species [19, 20], none of these are particularly well suited for large-scale production. The identification of the acetyltransferase *Ea*DAcT required for the synthesis of acetyl-TAGs in the seeds of *Euonymus alatus* has allowed the synthesis of these unusual molecules in species that typically do not produce them. For example, very high levels of acetyl-TAGs have been synthesized in the seeds of *Arabidopsis thaliana* and *Camelina sativa* by expressing *Ea*DAcT and simultaneously downregulating endogenous lcTAG production [14, 21]. Likewise, production of acetyl-TAGs has also been demonstrated in yeast. Here too, the elimination of regular lcTAG synthesis resulted in the accumulation of almost pure acetyl-TAGs [13] suggesting one strategy for the production of acetyl-TAGs. In this case, the removal of competing lcTAG biosynthetic enzymes was achieved by expressing *Ea*DAcT in a yeast background containing mutations in the *DGA1*, *LRO1*, *ARE1*, and *ARE2* genes that encode such activity [22].

Here, we demonstrate that acetyl-TAGs can be produced in a yeast strain previously engineered to use xylose as a carbon source. Further, we were able to increase the acetyl-TAG composition of the storage lipids produced in this strain by deleting endogenous enzymes known to contribute to lcTAG production. However, residual lcTAGs were still produced in these engineered yeast strains. Finally, we show that acetyl-TAGs can be synthesized by these strains when grown on a variety of carbon sources. Notably, acetyl-TAGs could be produced from pure xylose and AFEX corn stover hydrolysate (ACSH), the first demonstration that an advanced biofuel lipid can be synthesized from lignocellulose.

## Results

### The H1246 yeast mutant fails to produce TAGs when grown in corn stover hydrolysate

**Fig. 2:**
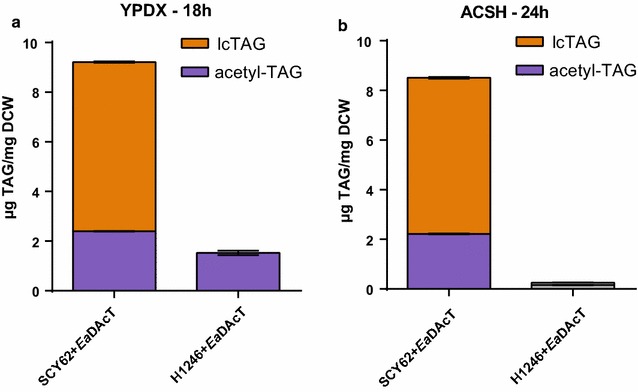
The H1246 yeast mutant fails to produce TAGs when grown in ACSH. Quantification of lcTAG and acetyl-TAG content of BY4741 background strains with different combinations of altered lcTAG biosynthesis or *Ea*DAcT expression grown on YPDX for 18 h (**a**) or ACSH for 24 h (**b**). Values represent the mean ± S.D. of acetyl-TAG or lcTAG content derived from four different ESI–MS analyses and are representative of at least two replicate cultures

Previously, we have demonstrated that yeast strains expressing *Ea*DAcT synthesize acetyl-TAGs from lab medium containing glucose [13]. In particular, expression of *Ea*DAcT in the H1246 mutant strain devoid of lcTAG synthesis [22] resulted in only acetyl-TAGs being present in the storage lipids. Desiring to synthesize acetyl-TAGs on sugar sources that would be readily available from lignocellulosic feedstocks, we cultured *Ea*DAcT expressing strains in AFEX corn stover hydrolysate (ACSH) and lab media containing yeast extract and peptone with glucose and xylose (YPDX) at equivalent concentrations to that found in ACSH. Similar to previous results when glucose was used [13], a mixture of lcTAGs and acetyl-TAGs was produced by the SCY62 wild-type background at similar titers in both YPDX and ACSH, with acetyl-TAGs constituting 26% of the total mass of TAGs produced (Fig. 2a; Table 1). The isogenic H1246 quadruple mutant expressing *Ea*DAcT, which does not synthesize lcTAGs [13], produced significant levels of acetyl-TAGs when grown on YPDX, but much lower titer and yield of acetyl-TAGs when grown in ACSH (Fig. 2b; Table 1). Also notable, the growth rates of SCY62 wild-type and H1246 mutant strains were lower in ACSH compared to YPDX media, resulting in 10- and 23-fold reductions, respectively, in the volumetric acetyl-TAG titers by the two strains between the two media conditions (Table 1). Importantly, no TAGs were identified from ACSH alone by thin-layer chromatography (data not shown), indicating that the yeast strains were producing acetyl-TAGs de novo, rather than taking up TAGs from the media.

**Table 1:** Fermentation properties for engineered and evolved *S. cerevisiae* strains

Medium	Aerobic YPDX			Aerobic ACSH		
Strain	SCY62+*Ea*DAcT	H1246+*Ea*DAcT	4KO+*Ea*DAcT	SCY62+*Ea*DAcT	H1246+*Ea*DAcT	4KO+*Ea*DAcT
Acetyl-TAG titer^a^	2400 ± 65	1500 ± 180	3000 ± 42	2200 ± 95	160 ± 19	1100 ± 6.2
Volumetric acetyl-TAG titer^b^	15 ± 2.0	6.9 ± 0.11	32 ± 2.5	14 ± 0.13	0.30 ± 0.036	7.9 ± 0.087
lcTAG titer^c^	6800 ± 0.20	ND	4200 ± 44	6300 ± 0.10	96 ± 1.1	2300 ± 2.2
Volumetric lcTAG titer^d^	43 ± 5.9	ND	45 ± 3.3	4.0 ± 0.37	0.18 ± 0.016	16 ± 0.10
% acetyl-TAGs^e^	31 ± 0.21	100 ± 0	48 ± 0.52	31 ± 0.097	67 ± 0.99	38 ± 0.36
Estimated growth rate^f^	0.34 ± 0.049	0.25 ± 0.035	0.58 ± 0.044	0.021 ± 0.0004	0.074 ± 0.002	0.29 ± 0.0003
Y_acTAG/glc_^g^	260 ± 9.5	130 ± 9.8	510 ± 48	120 ± 18	8.0 ± 1.5	120 ± 0.67
Y_lcTAG/glc_^h^	750 ± 32	ND	710 ± 65	330 ± 51	4.8 ± 0.76	250 ± 2.6

*ND* not detected

^a^In μg of acetyl-TAG/g of dry cell weight (DCW)

^b^In mg of acetyl-TAG/L

^c^In μg of lcTAG/g of DCW

^d^In mg of lcTAG/L

^e^Mole percentage of acetyl-TAG produced out of the total TAG produced

^f^In g of DCW/L/h within 18 h (in YPDX medium) or 24 h (in ACSH medium) of growth

^g^Yield in μg of acetyl-TAG produced/g of glucose consumed

^h^Yield in μg lcTAG produced/g of glucose consumed

### A wild yeast strain integrated with *Ea*DAcT produces acetyl-TAGs

**Fig. 3:**
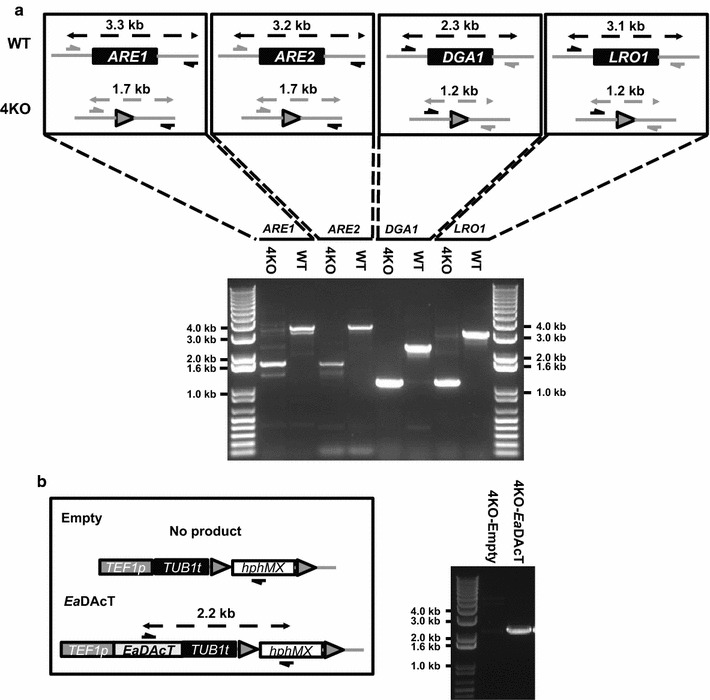
Engineering of acetyl-TAG production in a wild *S. cerevisiae* strain. A haploid version of the xylose-metabolizing GLBRCY2A strain was engineered for reduced lcTAG production. The schematic diagram in the *upper subpanel* of (**a**) indicates the predicted sizes (in kb) of PCR products generated by primers specific for wild-type *ARE1*, *ARE2*, *DGA1,* and *LRO1* or their corresponding marker-rescued deletions. Actual PCRs from the Y40 (WT) or Y40 strain containing all four deletions (4KO) are indicated alongside flanking DNA standards in the lower subpanel. PCR-verification of *Ea*DAcT insertion into the genome of 4KO is shown in (**b**). A schematic diagram showing predicted products from the primers used is on the *left*. Actual PCR confirmation in the indicated strains is shown on the* right*

Our observation that the H1246+*Ea*DAcT strain grew more slowly and produced acetyl-TAGs at lower yield in ACSH compared to YPDX (Table 1) suggested that components in ACSH had negative impacts on this strain background. ACSH contains significant concentrations of small molecules that are generated during biomass pretreatment, including phenolic acids, amides, aldehydes, organic acids, and acetamide [23–25] that impair growth and conversion of sugars into ethanol. One possible mechanism for reduced acetyl-TAG production is that the H1246+*Ea*DAcT strain was more sensitive to these toxic compounds present in ACSH. Previously, we identified a wild *S. cerevisiae* diploid strain, NRRL YB-210, with tolerance to inhibitors in ACSH and other stress-inducing conditions [26–28]. When engineered to express xylose reductase (*XYL1*), xylitol dehydrogenase (*XYL2*), and xylulokinase (*XYL3*) from *Scheffersomyces stipitis*, derivatives of this strain (GLBRCY2A) were able to convert xylose from synthetic lab media and ACSH into ethanol [27]. One haploid derivative of GLBRCY2A, named GLBRCY40 (referred to as Y40 from hereon), was selected for additional modification to produce acetyl-TAGs. First, we deleted *DGA1*, *LRO1*, *ARE1,* and *ARE2* coding sequences from the genome of Y40 (4KO; Fig. 3), and then inserted an empty or *Ea*DAcT-containing expression cassette into its genome (Fig. 3).

**Fig. 4:**
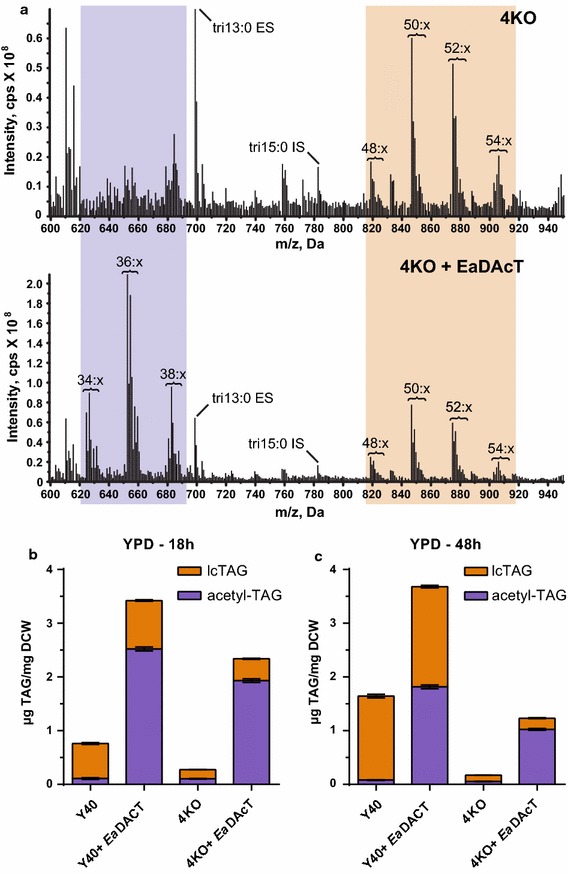
Xylose-enabled yeast strains expressing *Ea*DAcT produce acetyl-TAGs. **a** ESI mass spectra of neutral lipid extracts from xylose-enabled yeast strains where known enzymes for TAG biosynthesis were knocked out and *Ea*DAcT expressed after growth on YPD medium for 18 h. Acetyl-TAGs are highlighted in *purple* and lcTAGs in *orange*. Signal peaks possess the *m*/*z* value of the [M + NH_4_]^+^ adduct. For clarity, only the number of acyl carbons and not the number of double bonds (x) in each series of TAG molecular species is indicated. Tritridecanoin (tri13:0) is used as an ESI–MS external standard and tripentadecanoin (tri15:0) was added as an internal standard during lipid extraction. Quantification of lcTAG and acetyl-TAG content of different xylose-enabled yeast strains after 18 h (**b**) and 48 h (**c**) growth on YPD medium. Strains were either capable of normal lcTAG biosynthesis (WT) or possessed mutations in key biosynthetic genes (4KO) and had either *Ea*DAcT or control sequence integrated. Values represent the mean ± SD of acetyl-TAG or lcTAG content derived from four different ESI–MS analyses and are representative of at least two replicate cultures

We first sought to determine whether our strain engineering had effects on TAG production. Deletion of *DGA1*, *LRO1*, *ARE1,* and *ARE2* in our strain background did not significantly affect the cell growth on YPD aerobically (Additional file 1: Figure S1); this was also seen for other strain backgrounds possessing the same mutations [22]. Y40 and 4KO strains expressing *Ea*DAcT (Y40+*Ea*DAcT and 4KO+*Ea*DAcT, respectively) also grew to similar maximum cell densities (Additional file 1: Figure S1). To determine whether the engineered strains were capable of synthesizing acetyl-TAGs, we isolated neutral lipids from the cultured cells and analyzed them with electrospray ionization mass spectrometry (ESI–MS). In both the Y40 and 4KO backgrounds, peaks corresponding to the [M + NH_4_]^+^ masses of acetyl-TAG molecular species were detected in *Ea*DAcT-integrated lines at both 18 and 48 h of culture growth (Fig. 4a). In Y40+*Ea*DAcT, acetyl-TAGs comprised 78.5 mol% of the TAG content at 18 h; this level decreased to 56.0 mol% after 48 h of growth, even though the total amount of TAG remained the same (Fig. 4b). Importantly, deletion of the lcTAG-synthesizing enzymes increased the relative composition of acetyl-TAGs to 86 mol% at both 18 and 48 h in yeast expressing *Ea*DAcT (Fig. 4b). Surprisingly, small but significant amounts of lcTAGs were also detected in the 4KO strains (Fig. 4a). Despite expression of *Ea*DAcT, reduction of lcTAG synthesis also lowered the overall TAG content (including acetyl-TAGs) compared to the Y40+empty strain. This was particularly evident by 48 h when the total TAG content was 1.2 µg/mg DCW in the 4KO+*Ea*DAcT strain compared to 3.6 µg/mg DCW from the Y40+*Ea*DAcT strain. Together, these results confirm that the 4KO+*Ea*DAcT strain produces enriched amounts of acetyl-TAGs, but is not completely deficient for lcTAG production.

### Acetyl-TAGs can be produced when xylose is used as a carbon source

**Fig. 5:**
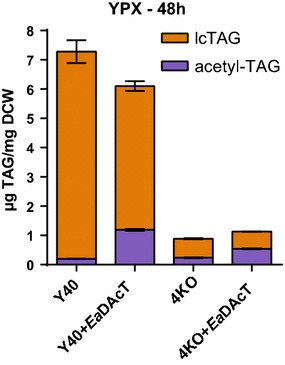
Acetyl-TAGs can be produced from xylose when used as a carbon source. Quantification of lcTAG and acetyl-TAG content of xylose-enabled strains with different combinations of altered lcTAG biosynthesis or *Ea*DAcT expression grown on YPX for 48 h. Values represent the mean ± SD of acetyl-TAG or lcTAG content derived from four different ESI–MS analyses and are representative of at least two replicate cultures

The primary goal of this work was to determine if acetyl-TAGs could be produced from ACSH. At 6% glucan loading, ACSH contains approximately 30 g/L of xylose and represents almost a third of the total sugars. Thus, we first determined whether acetyl-TAGs could be produced from xylose in lab media. In general, xylose was utilized at a slower rate compared to glucose by all strains, with significant amounts remaining in the media after 48 h and resulting in slower growth (Additional file 1: Figure S2) compared to what was seen with glucose (Additional file 1: Figure S1). While acetyl-TAGs were produced under these conditions, their levels were considerably lower compared to growth with glucose (Fig. 5). For example, in the 4KO+*Ea*DAcT strain, acetyl-TAGs were reduced to 54.6 mol% at 48 h (Fig. 5) compared to the 86 mol% composition observed after growth on YPD (Fig. 4b). These lower acetyl-TAG levels were due to not only reductions in the absolute quantities of acetyl-TAGs, but also to increased amounts of lcTAGs produced. Such increases were particularly evident in the Y40+empty and Y40+*Ea*DAcT strains, which produced 7.0 and 4.9 µg lcTAG/mg DCW, respectively (Fig. 5).

### Production of acetyl-TAG from lignocellulose-derived ACSH

**Fig. 6:**
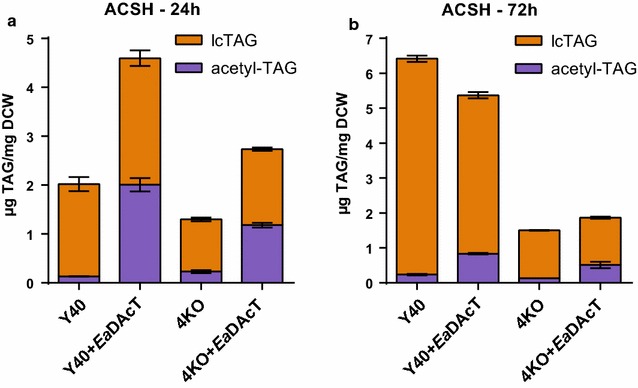
Acetyl-TAGs are produced from engineered yeast grown on ACSH. Quantification of lcTAG and acetyl-TAG content of xylose-enabled strains with different combinations of altered lcTAG biosynthesis or *Ea*DAcT expression grown on ACSH for 24 (**a**) or 72 (**b**) h. Values represent the mean ± SD of acetyl-TAG or lcTAG content derived from four different ESI–MS analyses and are representative of at least two replicate cultures

We next compared the ability of the 4KO+*Ea*DAcT strain to grow and produce acetyl-TAGs in YPDX and ACSH media. When grown on YPDX (with glucose and xylose at equivalent concentrations to that found in ACSH), the 4KO+*Ea*DAcT strain accumulated 3.0 µg acetyl-TAG/mg DCW, twofold higher than the mutant H1246+*Ea*DAcT strain, but also accumulated higher levels of lcTAGs (Table 1). The 4KO+*Ea*DAcT strain produced less acetyl-TAGs in ACSH compared to YPDX (Fig. 6; Table 1), but generated over sixfold more acetyl-TAGs on a cell mass basis than the H1246+*Ea*DAcT strain identically grown in ACSH. Importantly, the 4KO+*Ea*DAcT grew at an almost fourfold faster rate in ACSH, resulting in a 24-fold higher volumetric acetyl-TAG titer than the H1246+*Ea*DAcT strain (Table 1). While the SCY62+*Ea*DAcT strain produced twofold higher titer of acetyl-TAGs on a per cell mass basis (2.2 mg/g DCW) than the 4KO+*Ea*DAcT strain in ACSH, it also grew more slowly and therefore generated an almost sixfold lower volumetric titer (1.4 mg/L) than the 4KO+*Ea*DAcT strain (7.9 mg/L; Table 1). This supports the idea that the 4KO strain background is more tolerant to inhibitors in ACSH than both the SCY62 and H1246 strains. These differences also translated into an improved acetyl-TAG composition of 48 mol % in the total TAG extracted from the 4KO+*Ea*DAcT strain, compared to 31 mol% for the original SCY62+*Ea*DAcT strain. In stationary phase (72 h) after glucose was completely consumed and when xylose and ethanol could be respired (Additional file 1: Figure S3), acetyl-TAG levels in the Y40 and 4KO strains significantly dropped to 19 and 32 mol%, respectively (Fig. 6b). Similar to the situation with YPX, high levels of lcTAGs were observed, particularly in the Y40 strain where 6.1 and 4.5 µg lcTAG/mg DCW were produced in control and *Ea*DAcT-expressing cultures, respectively. Higher levels of lcTAGs were also observed in the 4KO background when grown on ACSH compared to YPD, but these were reduced compared to the Y40 strains (Figs. 4b, 6).

## Discussion

Expression of the DAG acetyltransferase *Ea*DAcT has been shown to be necessary and sufficient for the production of acetyl-TAGs in different transgenic plants, as well as in yeast [13, 14, 21]. Acetyl-TAGs possess unique and useful properties compared to regular lcTAGs and therefore represent useful molecules for a future biobased economy. Production of acetyl-TAGs from renewable biomass could further enhance the economic return. To this end, we engineered a stress-tolerant *S. cerevisiae* strain to express *Ea*DAcT (4KO+*Ea*DAcT), which enabled the generation of acetyl-TAGs from glucose in ACSH (Fig. 6). This contrasted what was seen with the H1246 laboratory strain, which did not generate any acetyl-TAGs from ACSH (Fig. 2b) and grew at a slower rate than in YPDX. We also found that the 4KO+*Ea*DAcT strain grew faster than both SCY62 and H1246 strains, particularly in ACSH. This ultimately translated into a faster rate and higher volumetric titer of TAG production by the 4KO strain relative to the others. Although the 4KO+*Ea*DAcT produced acetyl-TAGs from xylose in lab media (Fig. 5), it was unclear whether the strain generated acetyl-TAGs from xylose in ACSH. The reduction in acetyl-TAG levels in the stationary phase could be caused by a faster rate of acetyl-TAG catabolism compared to slower or absent rate of acetyl-TAG production from xylose. This inability to produce significant amounts of TAGs from xylose in ACSH may have resulted from cellular stress incurred by toxins present in ACSH. The effects of these toxins in ACSH on xylose conversion into ethanol have been seen in bacteria [23, 29] and yeast [25, 30]. Thus, while we accomplished our goal to generate acetyl-TAGs from lignocellulose, additional work is needed to utilize all of the sugars present in plant feedstocks.

Deletion of the four acyltransferases that synthesize lcTAGs greatly increased the relative acetyl-TAG composition produced by the engineered organism (Fig. 4). However, expression of *Ea*DAcT failed to fully compensate for the elimination of these four enzymes. Under all media conditions, the 4KO+*Ea*DAcT strain always produced less total TAG than Y40 cells also expressing *Ea*DAcT. Further, when at stationary phase time points in both lab media (YPD and YPX) and ACSH, the 4KO+*Ea*DAcT strain produced less total TAG than Y40 alone (Figs. 4b, 5 and 6b). These results are similar to what has been observed in *Arabidopsis* where the low oil content of the *dgat1* mutant is not fully complemented by expression of *Ea*DAcT [14]. Likewise, in *Camelina sativa*, suppression of endogenous lcTAG synthesis is associated with reduced oil content, despite the presence of *Ea*DAcT [14, 21]. A number of non-exclusive mechanisms could explain why the expression of *Ea*DAcT is unable to fully compensate for the elimination of most lcTAG biosynthesis, leading to a reduction in overall TAG content. One possibility is that the inability to synthesize lcTAGs leads to reduced fatty acid biosynthesis and a subsequent decrease in overall TAG accumulation. Similar effects have been noted in transgenic seeds engineered to produce unusual fatty acids. In these cases, a bottleneck in moving these unusual fatty acids from where they are synthesized on phosphatidylcholine (PC) to storage in TAGs leads to reduced fatty acid biosynthesis [31]. Alternatively, lcTAG biosynthesis appears to be carefully coordinated in yeast, with different enzymes more important at various growth phases. For example, PDAT activity is more important for lcTAG synthesis during exponential phase whereas DGAT2 activity predominates in stationary phase [32]. It is therefore possible that *Ea*DAcT expression did not adequately match the coordinated response of up to four different promoters. Thus, the *Ea*DAcT enzyme might not have been synthesized at the right levels and with the right timing to match the supply of available substrate. Further work is therefore needed to better elucidate the complex regulation that governs TAG accumulation in yeast, as well as in other organisms.

When the yeast cells were in stationary phase, acetyl-TAG levels decreased at late time points in the cultures. This was evident when the cells were grown on YPD and ACSH (Figs. 4, 6). We have also observed similar results when *Ea*DAcT is expressed in the H1246 background [33]. Recent work has suggested that instead of being inert end product pools, storage lipids are quite metabolically active, with evidence for TAG remodeling [34]. Our observations are consistent with this idea. In the case of Y40, in which the endogenous lcTAG biosynthetic enzymes are present, acetyl-TAGs were replaced by lcTAGs by stationary phase (Figs. 4, 5, 6). In 4KO strains, acetyl-TAGs were removed but not replaced by lcTAGs. Optimizing the length of culture growth will therefore be important in maximizing acetyl-TAG production. In addition, future work could identify the presumed lipases responsible for the TAG turnover, in order to overcome the observed reductions in acetyl-TAG.

Contrary to what was seen in H1246 strain, the targeted deletion of the four genes encoding the enzymes responsible for lcTAG synthesis in *S. cerevisiae* Y40 background failed to completely eliminate the production of lcTAGs (Figs. 4, 6; Table 1). As the parent strain NRRL YB-210 is of a different genetic background than the *S. cerevisiae* quadruple knockout H1246 [22], it is possible that yet to be identified acyltransferases capable of synthesizing lcTAGs exist in this background. Similar situations have occurred when studying the synthesis of lcTAGs in other yeast species. For example, when elimination of the DGAT2 and PDAT orthologs in *Yarrowia lipolytica* failed to completely eliminate lcTAG production, a DGAT1 enzyme was found to be responsible for the residual activity [35]. Likewise, in *Rhodotorula glutinis*, a member of the soluble DGAT3 family synthesizes the lcTAGs found in this oleaginous yeast species [36]. LcTAGs were not detected in ACSH, and H1246 yeast expressing *Ea*DAcT failed to synthesize lcTAGs when grown on ACSH and YPDX, indicating that the lcTAGs detected were synthesized *de novo* from the Y40-engineered yeast strains.

## Conclusions

In conclusion, we were able to demonstrate that acetyl-TAGs could be synthesized in a yeast strain capable of growing on carbohydrates derived from lignocellulosic feedstocks at a faster rate than previously published strains. Deletion of four genes important for lcTAG synthesis in this background enhanced the purity of the acetyl-TAGs produced under all media conditions, but failed to completely eliminate the synthesis of this competing metabolite. Interestingly, lcTAG levels increased when grown on xylose-containing media. These results imply that this strain contains other enzymes capable of synthesizing lcTAGs and that additional work is needed to fully understand the synthesis of storage lipids when grown on different carbohydrate sources.

## Methods

### Media preparation

Standard yeast lab media (YP) were prepared with 10 g/L yeast extract, 20 g/L peptone and 50 mM phosphate buffer, pH 5.0 in double distilled H_2_O and sterile filtered. Solid plate media also contained 25 g/L agar. Lab media containing dextrose (YPD) or xylose (YPX) were prepared with 20 g/L dextrose or 20 g/L xylose, respectively, while YPDX media contained 60 g/L dextrose and 30 g/L xylose. AFEX corn stover hydrolysate (ACSH) from 6% glucan loading was prepared as described previously [30]. In brief, *Zea mays* (Pioneer hybrid 36H56) stover harvested in 2012 was AFEX pretreated and hydrolyzed with CTec2 and HTec2 enzymes (Novozymes). After 7 days, the hydrolysate was centrifuged and filtered. For strains transformed with plasmids, 200 μg/mL Hygromycin B (Life Technologies) was added to the media.

### *Saccharomyces cerevisiae* strain and *Ea*DAcT plasmid construction

Genotypes of *S. cerevisiae* strains used in this study are described in Additional file 1: Table S1. SCY62 and H1246 yeast strains have been described previously [22]. GLBRCY40 containing deletions of four known genes involved in lcTAG synthesis was generated from a haploid isolate of GLBRCY2A, a wild diploid *S. cerevisiae* strain engineered for xylose metabolism [27]. In brief, Y2A was sporulated in 1% potassium acetate for 10 days and individual tetrads dissected on YPD plates. Individual spores were then verified for a single mating type. One spore, named GLBRCY27D, was selected and subjected to *kanMX* marker rescue with pSH65 plasmid [37]. The resulting strain, Y40, was then transformed [38] with *loxP-kanMX-loxP* polymerase chain reaction (PCR) product amplified using Phusion polymerase (Thermo Fisher) and primers containing 45 bp DNA sequences flanking the *DGA1* open reading frame. Confirmation of gene deletion by homologous recombination was performed by PCR of genomic DNA. The *loxP-kanMX*-*loxP* marker was rescued as described above. This process was repeated for subsequent deletions of *LRO1*, *ARE2*, and *ARE1* to generate the Y40 *are1Δ*::*loxP are2Δ*::*loxP dga1Δ*::*loxP lro1Δ*::*loxP* quadruple knockout strain (4KO) with the *loxP*-KanMX marker rescued.

To generate acetyl-TAG-producing strains, the *Ea*DAcT open reading frame (ORF) was codon optimized for *S. cerevisiae* and synthesized (GeneArt). The synthetic *Ea*DAcT sequence was then inserted between the *S. cerevisiae TEF1* promoter and *TUB1* terminator of the pRS2μ-2gene plasmid. In brief, the pRS2μ-2gene plasmid contains 667 bp of the *ACT1* promoter next to 350 bp of the *TEF1* terminator, then 579 bp of the *TEF1* promoter with 569 bp of the *TUB1* terminator, followed by the *loxP*-*hphMX4*-*loxP* marker, inserted between the *Sac*I to *Kpn*I polylinker sites of pRS426 [39] lacking the *URA3* marker. To integrate the expression cassette into the Y40 wild-type (WT) and 4KO strains, *CYC1* terminator and HO-R [40] DNA sequences were inserted into the *Sac*I and *Kpn*I sites, respectively, of the pRS2μ-2gene empty or pRS2μ-2gene + *Ea*DAcT plasmids. T3 and T7 primers were used to PCR amplify the *CYC1* terminator-*ACT1* promoter-*TEF1* terminator-*TEF1* promoter-*Ea*DAcT ORF-*TUB1* terminator-*loxP*-*hphMX*-*loxP*-HO-R cassette between the pRS2μ plasmid multi-cloning sites, which was then transformed into the Y40 WT or 4KO strains. Genome insertion of this cassette was verified by PCR. For SCY62 and H1246 strains, the pRS2μ-2gene + *Ea*DAcT plasmid was transiently transformed and selected by addition of Hygromycin B to all media.

### Yeast culturing experiments

All yeast growth experiments were performed in four 2.5 L vessel Parallel Bioreactor systems (DASGIP) or six 200 mL mini-Bioreactors with myControl controllers (Applikon Biotechnology). Vessels were sparged with air at 0.13 L/min for aerobic experiments. Inocula were prepared from single colonies grown in YPD media overnight at 30 °C. Cells were then centrifuged, the resulting cell pellet washed with YP media without sugars, and then resuspended in the same media as in the cell culture experiment. Strains were then inoculated into bioreactors to achieve a starting cell density of optical density of 0.1 at *λ* = 600 nm in a 1-cm path length cuvette spectrophotometer (Beckman Coulter). Vessels were maintained at 30 °C and pH 5.0 by addition of NaOH or HCl and stirred with impeller speeds of 300 RPM. Cell densities during specified times of fermentation experiments were measured by optical density at 600 nm. Extracellular sugar concentrations were measured by a YSI 2700 instrument (YSI Inc.) and dry cell weight (DCW) determinations were performed as described elsewhere [30].

### Quantification of TAGs from yeast

At specified times, 25–50 mL of yeast culture was removed from bioreactors and dispensed into 50-mL conical tubes. Cells were harvested when glucose was nearly or completely depleted from the media [18 or 24 h after inoculation for YPDX or ACSH, respectively; the differences in harvest time were due to growth rate differences in the two different media conditions (see Table 1)]. Harvested cultures were then centrifuged at 10,000 RCF at 4 **°**C for 5 min. Clarified media were decanted, cells were washed twice in TE buffer (10 mM Tris, 1 mM EDTA, pH 7.0), and the final cell pellet flash-frozen in dry ice-ethanol. Total lipids were extracted and neutral lipids isolated as described previously [13]. 50 n moles of tripentadecanoin (Nu-Check Prep) was added to each sample prior extraction. To quantify acetyl-TAGs and lcTAGs using ESI–MS, 1 µg of neutral lipids dissolved in 400 µL chloroform was mixed with 700 µL of methanol:300 mM ammonium acetate, 100:5.26 (v/v). 3 µL of 10 µM tritridecanoin internal standard was spiked into every sample prior to ESI-MS analysis. Neutral lipids were analyzed in positive ion mode using a triple quadrupole mass spectrometer API 400 (Applied Biosystems) equipped with an ESI source. The samples were directly infused at 30 µL/min. Instrument setting for total ion scans was as follows: curtain gas, 20 (arbitrary units); ion source gases 1 and 2, 45 (arbitrary units), ion spray voltage, 5500 V; source temperature, 100 °C; declustering potential, 20 V; entrance potential, 10 V; and the interface heater, “on”. Spectra were acquired from 500 to 1000 *m/z* at 5 s per cycle for 40 cycles. Spectra were smoothed one time (3-point boxcar) with 0.4 for previous and next point weight and 1 for current point weight. The baseline was subtracted with a window of 20 u. Spectral data were processed and exported using the “MultiplePeriodProcessing” function provided by Analyst software (Applied Biosystems). Mass peaks corresponding to acetyl-TAG and lcTAG molecular species were deconvoluted for M + 2 and M + 4 isotopic overlap and corrected for isotopic variation using an inhouse script that utilizes the creation of isotopomer abundance matrixes [41]. Correction for the effect of the number of acyl chain carbons and double bonds on the signal strength was performed as previously described [42]. Acetyl-TAG and lcTAG abundances were normalized to that of the tripentadecanoin standard to correct for extraction efficiency.

## Additional file

**Additional file 1: Figure S1.** Elimination of lcTAG synthesis and expression of *Ea*DAcT does not affect growth on YPD. Cell density (C.D.; solid lines) and extracellular glucose concentrations (Glc; dashed lines) from indicated strains cultured aerobically in bioreactors containing YPD media. Values displayed are the mean ± S.D. from three independent biological replicates. **Figure S2.** Acetyl-TAG producing yeast strains can consume and grow on xylose. Cell density (C.D.; solid lines) and extracellular xylose concentrations (Xyl; dashed lines) from indicated strains cultured aerobically in bioreactors containing YPX media. Values displayed are the mean ± S.D. from three independent biological replicates. **Figure S3.** Acetyl-TAG producing yeast strains consume sugars in ACSH similarly to wild-type cells. Cell density (C.D.; solid lines) and concentrations of extracellular glucose (Glc; dashed lines) and xylose (Xyl; dashed lines) from indicated strains cultured aerobically in bioreactors containing ACSH. Values displayed are the mean ± S.D. from three independent biological replicates. **Table S1.** *S. cerevisiae* strains used in this study. **Table S2.** Primers used for genetic engineering.

## Abbreviations

acetyl-TAGs: acetyl-triacylglycerols
ACETEM: acetic acid esters of mono- and diglycerides
ACSH: AFEX-pretreated corn stover hydrolysate
AFEX: ammonia fiber expansion
ESI–MS: electrospray ionization mass spectrometry
lcTAGs: long chain triacylglycerols
TAG: triacylglycerol
YPD: yeast extract peptone dextrose
YPDX: yeast extract peptone dextrose xylose
YPX: yeast extract peptone xylose
