# Supplementary material for: Metabolic engineering of Saccharomyces cerevisiae to produce a reduced viscosity oil from lignocellulose
Source: Biotechnol Biofuels. 2017 Mar 20;10:69. doi: 10.1186/s13068-017-0751-y (PMC5359884; doi:10.1186/s13068-017-0751-y)
Supplement: Supplementary file 1 — Additional file 1: Figure S1. Elimination of lcTAG synthesis and expression of EaDAcT does not affect growth on YPD. Cell density (C.D.; solid lines) and extracellular glucose concentrations (Glc; dashed lines) from indicated strains cultured aerobically in bioreactors containing YPD media. Values displayed are the mean ± S.D. from three independent biological replicates. Figure S2. Acetyl-TAG producing yeast strains can consume and grow on xylose. Cell density (C.D.; solid lines) and extracellular xylose concentrations (Xyl; dashed lines) from indicated strains cultured aerobically in bioreactors containing YPX media. Values displayed are the mean ± S.D. from three independent biological replicates. Figure S3. Acetyl-TAG producing yeast strains consume sugars in ACSH similarly to wild-type cells. Cell density (C.D.; solid lines) and concentrations of extracellular glucose (Glc; dashed lines) and xylose (Xyl; dashed lines) from indicated strains cultured aerobically in bioreactors containing ACSH. Values displayed are the mean ± S.D. from three independent biological replicates. Table S1. S. cerevisiae strains used in this study. Table S2. Primers used for genetic engineering. [file 13068_2017_751_MOESM1_ESM.pdf]

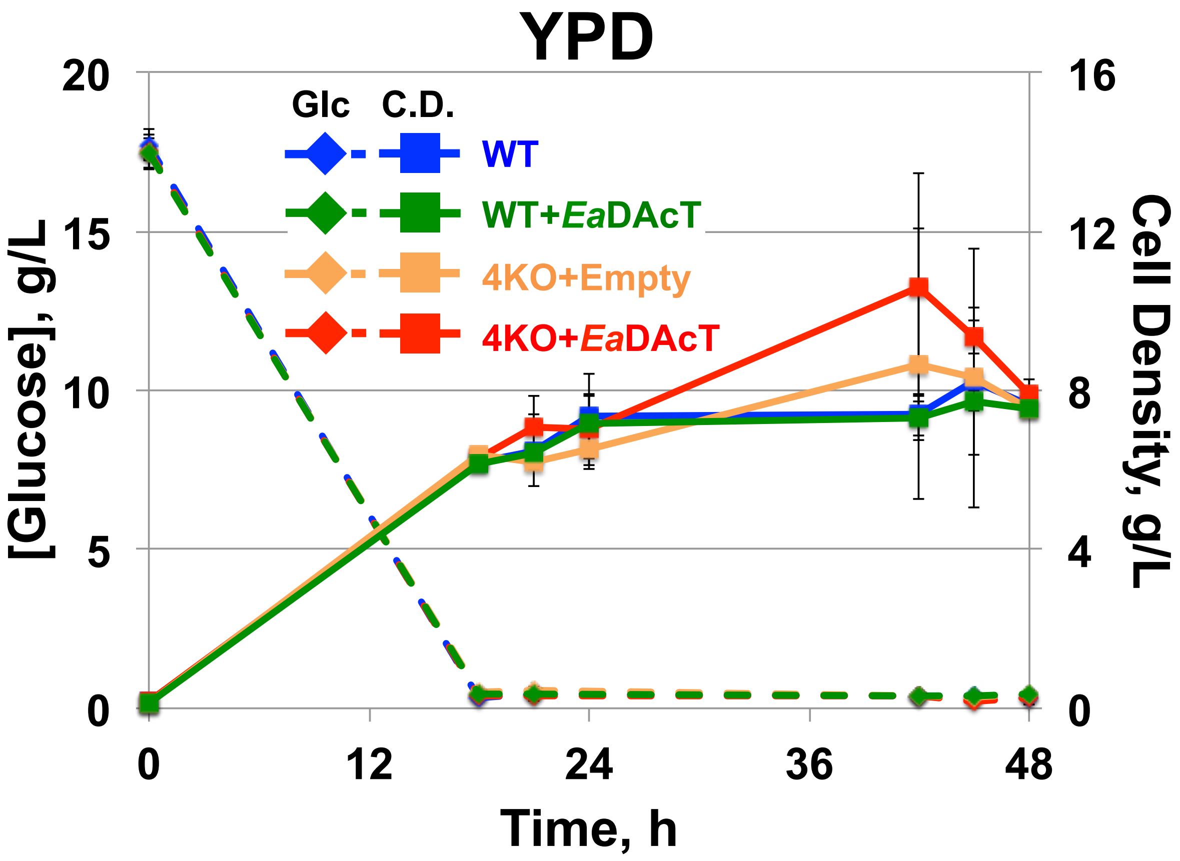

Figure S1. Elimination of lcTAG synthesis and expression of *Ea*DAcT does not affect growth on YPD. Cell density (C.D.; solid lines) and extracellular glucose concentrations (Glc; dashed lines) from indicated strains cultured aerobically in bioreactors containing YPD media. Values represent the mean ± S.D. and are representative of three biological replicates.

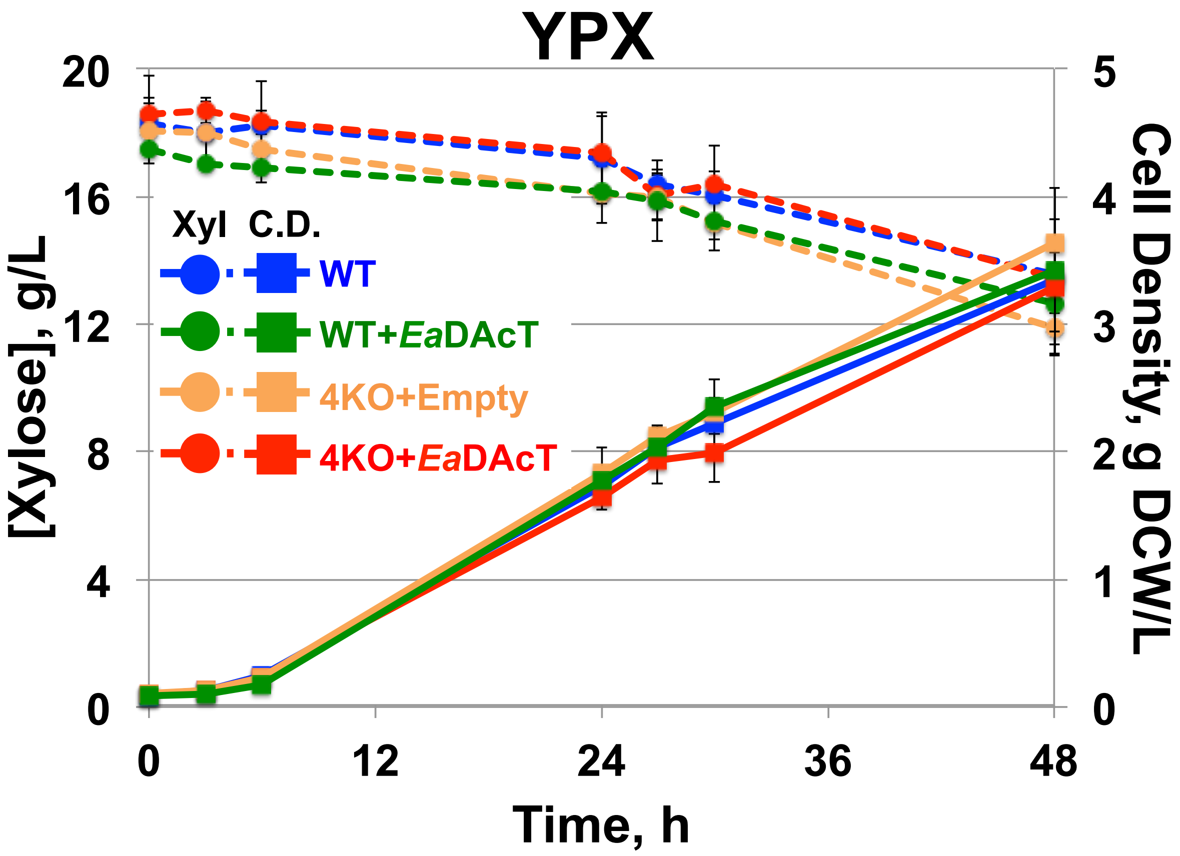

Figure S2. Acetyl-TAG producing yeast strains can consume and grow on xylose. Cell density (C.D.; solid lines) and extracellular xylose concentrations (Xyl, dashed lines) from indicated strains cultured aerobically in bioreactors containing YPX media. Values represent the mean ± S.D. and are representative of three biological replicates.

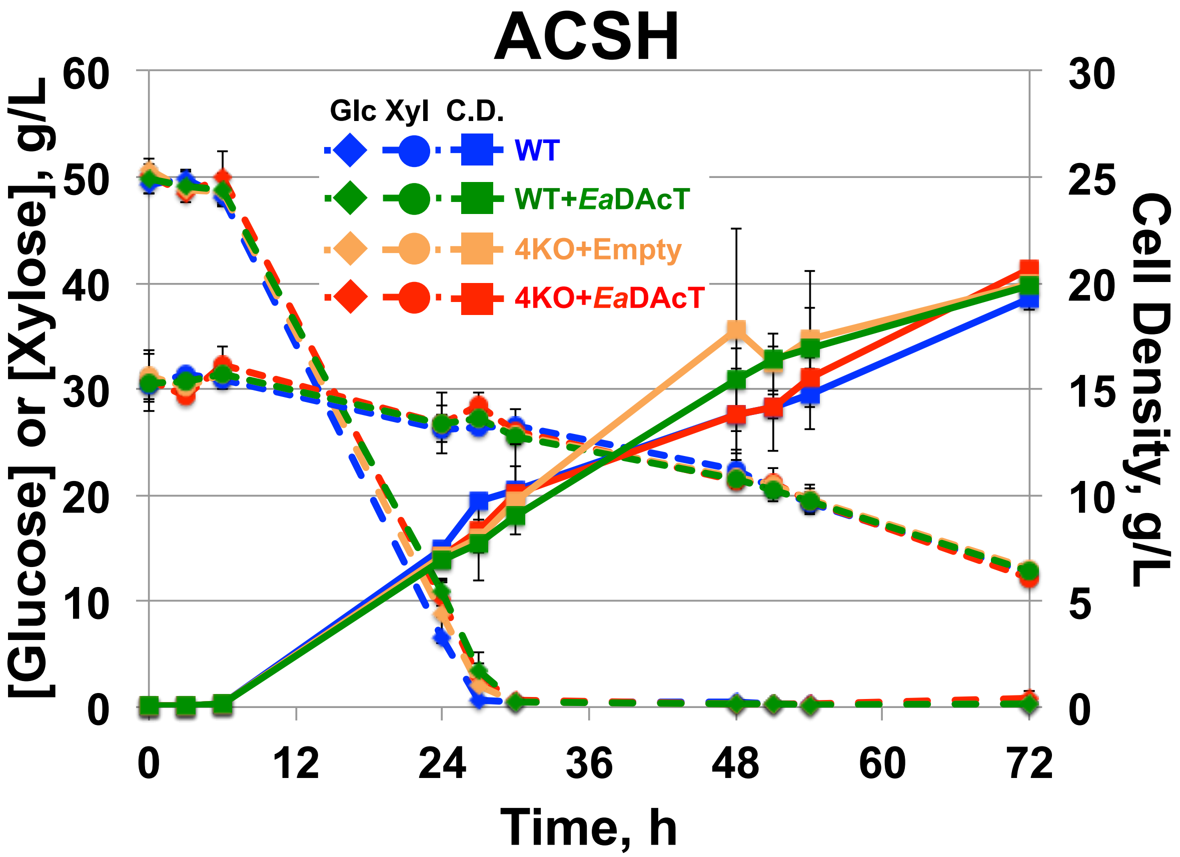

Figure S3. Acetyl-TAG producing yeast strains consume sugars in ACSH similarly to wild-type cells. Cell density (C.D.; solid lines) and concentrations of extracellular glucose (Glc; dashed lines) and xylose (Xyl; dashed lines) from indicated strains cultured aerobically in bioreactors containing ACSH. Values represent the mean ± S.D. and are representative of three biological replicates.

Table S1. *S. cerevisiae* strains used in this study.

Strain name	Genotype	Reference
SCY62	MATa *ADE2*	[21]
H1246	MATa *are1Δ::HIS3 are2Δ::LEU2 dga1Δ::KanMX4 lro1Δ::TRP1 ADE2*	[21]
GLBRCY2A	NRRL YB-210; MATα/MATa *HO/HOΔ::PGK1p-XYL1-TDH3t-TDH3p-XYL2-TEF2t-TEF2p-XYL3-CYC1t-LoxP-KanMX-LoxP*	[26]
GLBRCY27D	Haploid derivative of GLBRCY2A; MATα *HOΔ::PGK1p-XYL1-TDH3t-TDH3p-XYL2-TEF2t-TEF2p-XYL3-CYC1t-LoxP-KanMX-LoxP*	This study
WT, GLBRCY40	GLBRCY27D with *loxP-KanMX-loxP* marker excised by Cre	This study
4KO, GLBRCY375	GLBRCY40 *are1Δ::LoxP are2Δ::LoxP dga1Δ::LoxP lro1Δ::LoxP*	This study
WT+EaDAcT, GLBRCY123	GLBRCY40 *HOΔ::PGK1p-XYL1-TDH3t-TDH3p-XYL2-TEF2t-TEF2p-XYL3-CYC1t -ACT1p-TEF1t-TEF1p-EaDAcT-TUB1t-LoxP-HphMX4-LoxP*	This study
4KO+Empty, GLBRCY122	GLBRCY375 *HOΔ::PGK1p-XYL1-TDH3t-TDH3p-XYL2-TEF2t-TEF2p-XYL3-CYC1t -ACT1p-TEF1t-TEF1p-TUB1t-LoxP-HphMX4-LoxP*	This study
4KO+EaDAcT, GLBRCY121	GLBRCY375 *HOΔ::PGK1p-XYL1-TDH3t-TDH3p-XYL2-TEF2t-TEF2p-XYL3-CYC1t -ACT1p-TEF1t-TEF1p-EaDAcT-TUB1t-LoxP-HphMX4-LoxP*	This study

In the genotype description, "*p*" denotes promoter sequence and "*t*" denotes terminator sequence.

Table S2. Primers used for genetic engineering.

Primer name	Purpose	Sequence (5' to 3')
ybDGA1LoxPFOR	Forward primer to amplify LoxP-KanMX4-LoxP cassette for deletion of *DGA1* ORF	ATACATAAGGAAACGCAGAGG CATACAGTTTGAACAGTCA CATAA agctgaagcttcgtacgctgc
ybDGA1LoxPREV	Reverse primer to amplify LoxP-KanMX4-LoxP cassette for deletion of *DGA1* ORF	CCTTATTTATTCTAACATATTTTGT GTTTTCCGATGAATTCATTA gcataggccactagtggatctg
ybLRO1LoxPFOR	Forward primer to amplify LoxP-KanMX4-LoxP cassette for deletion of *LRO1* ORF	ACAAAAGGTTCTCTACCAACGA ATTCGGCGACAATCGAGT AAAAA agctgaagcttcgtacgctgc
ybLRO1LoxPREV	Reverse primer to amplify LoxP-KanMX4-LoxP cassette for deletion of *LRO1* ORF	TCTTTGAAATAATACACGGAT GGATAGTGAGTCAATGTCG GTCAT gcataggccactagtggatctg
ybARE1LoxPFOR	Forward primer to amplify LoxP-KanMX4-LoxP cassette for deletion of *ARE1* ORF	TGTTCAGCACGGCTTGCAGC AAGAGCGCCAAAACAGATT GCAAGA agctgaagcttcgtacgctgc
ybARE1LoxPREV	Reverse primer to amplify LoxP-KanMX4-LoxP cassette for deletion of *ARE1* ORF	TATCTATCAAGGGCTTGCGAG GGACACACGTGGTACGGTG GCAGT gcataggccactagtggatctg
ybARE2LoxPFOR	Forward primer to amplify LoxP-KanMX4-LoxP cassette for deletion of *ARE2* ORF	CAAGGACACATTACGTTAGC AAAAGCAACAATAACAAACA CAACC agctgaagcttcgtacgctgc
ybARE1LoxPREV	Reverse primer to amplify LoxP-KanMX4-LoxP cassette for deletion of *ARE2* ORF	CTCCACAGAACAGTTGCAGG ATGCCTTAGAATGTCAAGTAC AACG gcataggccactagtggatctg
ScARE1-507FOR	Forward primer to verify deletion of *ARE1*, anneals 507 bp upstream of *ARE1* ORF	gctccaacgccctcagcgtc
ScARE1+998REV	Reverse primer to verify deletion of *ARE1*, anneals 998 bp downstream of *ARE1* ORF	agagcctttgtgctgggtcg
ScARE2-515FOR	Forward primer to verify deletion of *ARE2*, anneals 515 bp upstream of *ARE2* ORF	gcgccgctgggaaaacgcac
ScARE2+986REV	Reverse primer to verify deletion of *ARE2*, anneals 986 bp downstream	ctttttggcgcgttttcgcc

	of *ARE2* ORF	
ScDGA1-541FOR	Forward primer to verify deletion of *DGA1*, anneals 541 bp upstream of *DGA1* ORF	caatgtcgacgaagcggcatcg
ScDGA1+503REV	Reverse primer to verify deletion of *DGA1*, anneals 503 bp downstream of *DGA1* ORF	cctatagagcctattggtcgg
ScLRO1-534FOR	Forward primer to verify deletion of *LRO1*, anneals 534 bp upstream of *LRO1* ORF	cgacacattgctcggcacg
ScLRO1+520REV	Reverse primer to verify deletion of *LRO1*, anneals 520 bp downstream of *LRO* ORF	gctcctcattaggggaacagg

Primer sequences in upper case denote sequences used for homologous recombination; lower case denotes annealing sequences.
